# High-Throughput Analysis of Global DNA Methylation Using Methyl-Sensitive Digestion

**DOI:** 10.1371/journal.pone.0163184

**Published:** 2016-10-17

**Authors:** Hiromi Shiratori, Carmen Feinweber, Claudia Knothe, Jörn Lötsch, Dominique Thomas, Gerd Geisslinger, Michael J. Parnham, Eduard Resch

**Affiliations:** 1 Project Group Translational Medicine and Pharmacology TMP, Fraunhofer Institute for Molecular Biology and Applied Ecology IME, Frankfurt am Main, Germany; 2 Institute of Clinical Pharmacology, Goethe - University, Frankfurt am Main, Germany; Inc, UNITED STATES

## Abstract

DNA methylation is a major regulatory process of gene transcription, and aberrant DNA methylation is associated with various diseases including cancer. Many compounds have been reported to modify DNA methylation states. Despite increasing interest in the clinical application of drugs with epigenetic effects, and the use of diagnostic markers for genome-wide hypomethylation in cancer, large-scale screening systems to measure the effects of drugs on DNA methylation are limited. In this study, we improved the previously established fluorescence polarization-based global DNA methylation assay so that it is more suitable for application to human genomic DNA. Our methyl-sensitive fluorescence polarization (MSFP) assay was highly repeatable (inter-assay coefficient of variation = 1.5%) and accurate (r^2^ = 0.99). According to signal linearity, only 50–80 ng human genomic DNA per reaction was necessary for the 384-well format. MSFP is a simple, rapid approach as all biochemical reactions and final detection can be performed in one well in a 384-well plate without purification steps in less than 3.5 hours. Furthermore, we demonstrated a significant correlation between MSFP and the LINE-1 pyrosequencing assay, a widely used global DNA methylation assay. MSFP can be applied for the pre-screening of compounds that influence global DNA methylation states and also for the diagnosis of certain types of cancer.

## Introduction

DNA cytosine methylation at position 5 in the pyrimidine ring (5mC) is predominantly observed in the context of CpG dinucleotides in human [1]. It is a hallmark of transcriptional gene silencing and heterochromatin formation in conjunction with chromatin remodelling factors [2–5]. It also plays crucial roles in key physiological processes, including differentiation and chromosome stability [6–8]. Aberrant DNA methylation patterns and global DNA hypomethylation are associated with various diseases, including developmental disorders and cancer [1, 9–13].

Compounds such as 5-azacytidine and bisphenol A can deregulate gene expression and cause genomic instability by interfering with DNA methylation states [1, 14–16]. Therefore, drugs with epigenetic effects have received intensive interest for clinical application [11, 17, 18]. Some natural and chemically synthesized pharmaceutical compounds, however, have demonstrated unexpected epigenetic effects [15]. These recent studies highlight the importance of measuring genome-wide CpG methylation, but the high-throughput capacity of most methods for the detection of global DNA methylation, for instance LINE-1 pyrosequencing, is limited to the 96-well format (Table 1). Classical 5mC quantification assays using liquid chromatography, mass spectrometry, gel electrophoresis, and capillary electrophoresis are considered as gold standards, but they are unsuitable for the simultaneous analysis of multiple samples [19–22]. Whole genome bisulfite sequencing, a bisulfite conversion of genomic DNA combined with next-generation sequencing, is a powerful technology to investigate genome-wide DNA methylation profiles with single-base resolution [23, 24], though the method is costly and requires substantial bioinformatics [25]. Despite the advent of novel approaches based on next-generation sequencing, methylation-sensitive restriction enzymes that differentiate between methylated and unmethylated CpG dinucleotides are still widely used for the quantitative analysis of genome-wide methylation [26–28]. Restriction enzyme-based approaches have been developed mainly for the 96-well format, although these are time consuming and expensive and therefore unsuitable for large-scale screening applications (Table 1).

**Table 1:** Comparison of high-throughput global DNA methylation assays.

Assay	Approx assay duration	DNA (ng)	Features/requirements	Internal control	Resolution	Format	Reference
**MSFP**	3.5 h	10–80	All organisms No enrichment/purification RE-based method	Included	CpG within RE sites	384-well	[29]
**LUMA**	5 h	100–500				96-well	[30–32]
**LINE-1**	5.5 h	2–166	Organism-specific due to primers (LINE-1: eukaryotes, Alu: primates) Enrichment required for pyrosequencing Bisulfite-conversion of DNA Pyrosequencing or MethyLight qPCR	Pyro- sequencing: not required MethyLight qPCR: included	CpG within LINE-1/Alu		[31–35]
**Alu**	5.5 h	2–25					[31, 32]
**CpGlobal**	2 d	100	All organisms Enrichment required RE-based method	Included	CpG within RE sites		[19]
**ELISA**	3–4 h	100	All organisms Multiple washing steps required	No	Over-all 5mC		[36–38]

The table focuses on assay conditions, methods, and readouts downstream of the DNA purification step. Internal controls are used to normalize the input DNA amount. Abbreviations: 5mC; 5-methylcytosine, LINE-1; long interspersed nuclear element-1, LUMA; luminometric methylation assay, MSFP; methyl-sensitive fluorescence polarization, RE; restriction enzyme.

Fluorescence polarization (FP) can be used to quantify molecular interactions, enzyme activity, and nucleic acid hybridization [39–41], and has more recently been extended to investigate global DNA methylation [29]. FP is ideal for high-throughput screening, as reflected in its reproducibility, small sample volumes, rapid and easy handling without purification steps, and the availability of 384-well assay formats [40, 42]. The principle of FP is the inverse correlation between the polarization degree of a fluorophore and its molecular rotation. When a small fluorescent-labelled molecule is excited with linearly-polarized light, the emitted light is largely depolarized due to the rapid molecular rotation of the fluorophore during its fluorescent lifetime, whereas when the small molecule is bound to a larger target it is stabilized and rotates slowly, so that the emitted light remains polarized.

In this study, we have improved the previously established FP-based global DNA methylation assay [29] making it more suitable for applications involving human genomic DNA. We also confirmed the significant correlation between the results achieved using this approach and the gold standard LINE-1 pyrosequencing assay which is widely used for the analysis of global DNA methylation.

## Results

**Fig 1:**
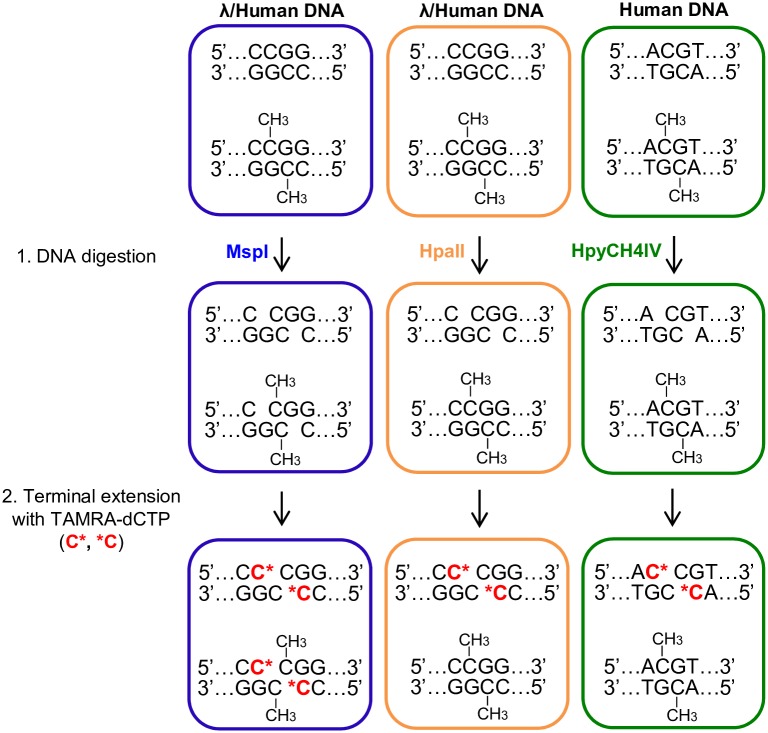
The workflow of the methyl-sensitive fluorescence polarization (MSFP) assay. Lambda (λ) and human genomic DNA are restricted by MspI (white) or by HpaII each alone or by a combination of HpaII and HpyCH4IV (grey) (Step 1). Subsequently, digested DNA with CpG overhangs at the 5' termini are terminally extended with fluorescence-labelled TAMRA-dCTP (C*, *C) (Step 2). TAMRA-dCTP incorporated into DNA is quantified by fluorescence polarization directly on the plate without additional purification procedures.

We established a methyl-sensitive fluorescence polarization (MSFP) assay based on the previously described method [29] by incorporating the unique modifications summarized in Fig 1. The restriction endonucleases MspI and HpaII are isoschizomers that recognize the palindromic target sequence CCGG, but HpaII is unable to cleave the site when the inner CG dyad is fully- or hemi- methylated [43, 44], making the MspI-HpaII pair a valuable tool for methylation analysis. In order to increase the signal output, another methylation-sensitive restriction enzyme (HpyCH4IV, target sequence ACGT) was introduced in addition to HpaII for application to human genomic DNA (Step 1). All the restriction enzymes we used generate a CpG overhang at the 5'-terminus on both complementary strands, serving as templates during the sequential terminal extension step with TAMRA-dCTP, a fluorescent labelled cytosine (Step 2). The incorporation of TAMRA-dCTP into the digested DNA can be quantified using a fluorescence polarization (FP) assay without any additional purification and enrichment procedures. In principle, FP detects the binding of small fluorescent molecules to larger objects because fluorescent labelled molecules excited by polarized light emit light with a degree of polarization that is inversely proportional to the rate of molecular rotation [40]. Unbound TAMRA-dCTP, therefore, produces low background FP, whereas the same molecule incorporated into DNA yields a stronger FP signal. The FP signal of samples digested with MspI (FP_M_) provides the internal reference for the amount of DNA, whereas the signal from samples digested with HpaII or the combination HH (FP_H_ and FP_HH_, respectively) indicate the content of methylated cytosine (5mC). Accordingly, the quantity of 5mC in a sample can be calculated by normalizing the FP_H_ and FP_HH_ values (5mC indicators) respectively, to the FP_M_ value.

### Verification of the MSFP assay

**Fig 2:**
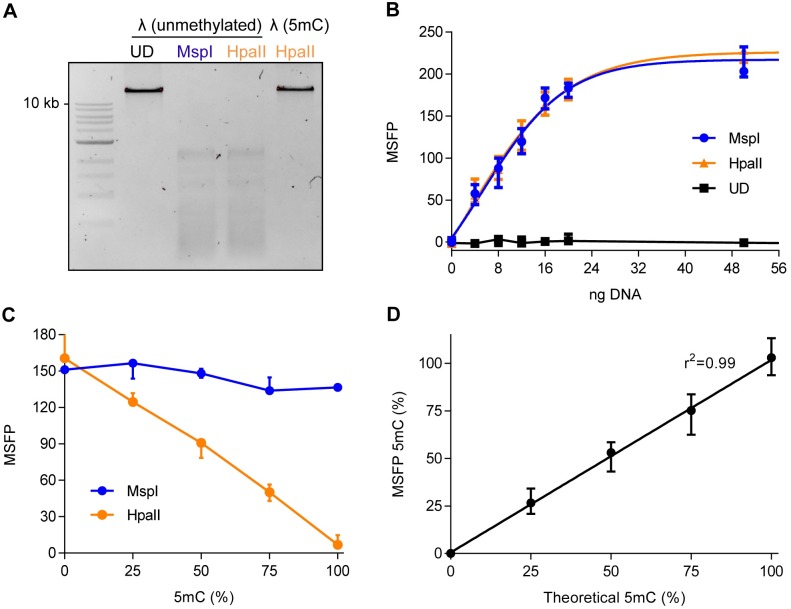
Measurement of global DNA methylation using the MSFP assay. (A) In-vitro methylation of λ DNA was confirmed by HpaII digestion. Unmethylated and fully-methylated (5mC) λ DNA were digested by either MspI or HpaII and the digestion pattern was visualized by agarose gel electrophoresis. UD: undigested. (B) The standard curves represent unmethylated λ DNA, showing medians and ranges of 3–7 independent experiments, each performed in technical duplicates or triplicates. The amounts of digested DNA applied per well are indicated on the x-axis. (C) The MSFP measurement of λ DNA with different DNA methylation levels. Unmethylated and 5mC λ DNA were mixed to obtain 10 ng of DNA with various DNA methylation levels (0, 25, 50, 75, 100%) followed by the MSFP assay. The data show medians and ranges of three independent experiments, each performed in technical triplicates. (D) The accuracy of the DNA methylation level (%) determined by the MSFP. The DNA methylation content (5mC) of each analyte from Fig 2C was calculated according to the formula in the Materials and Methods section. The data represent medians and ranges of three independent experiments, each performed in technical triplicates.

The MSFP assay was verified using lambda (λ) DNA, which is 48,502 bp in length and and is not methylated at CpG sites (manufacturer’s information). Fully-methylated λ DNA can be obtained by incubation with M.SssI, a CpG methyltransferase that adds a methyl group to cytosine residues in the CpG motif. The testing of λ DNA with a defined global DNA methylation content allowed us to determine the specificity and sensitivity of the system. First, complete in-vitro methylation of λ DNA was confirmed by HpaII digestion and gel electrophoresis (Fig 2A). As expected, we observed similar digestion patterns when unmethylated λ DNA was treated with MspI and HpaII. In contrast, fully-methylated (5mC) λ DNA was not digested by HpaII yielding a single band identical to that in the undigested control lane, confirming that all MspI/HpaII recognition sites of 5mC λ DNA were fully-methylated. Furthermore, we confirmed the complete in vitro methylation of λ DNA using LC-MS/MS analysis (S1 Fig, S1 Table and S1 Protocol). Next, standard curves were generated to determine the correlation between the quantity of DNA and the FP signal, thus, defining the linear range of the MSFP assay (Fig 2B). The quantity of digested DNA was positively correlated with FP signal strength, but the FP signal representing the undigested DNA remained at the baseline level regardless of the amount of DNA. This indicated that the FP signal is specific for TAMRA-dCTP incorporated into DNA and that the quantity of DNA does not interfere with the assay. Notably, we found that the strength of the FP signal increased linearly at low DNA concentrations but reached saturation when the amount of DNA exceeded 20 ng. We then prepared λ DNA with different levels of 5mC by mixing unmethylated and 5mC λ DNA, and the extent of DNA methylation was measured using the MSFP assay. Standard curves were included in all experiments to ensure that all FP values fell within the linear range. All MspI-digested samples yielded an FP signal of ~150, indicating that each analyte contained a similar amount of DNA (Fig 2C). In contrast, the HpaII-digested samples yielded a signal that declined in line with the increasing 5mC content. In agreement with the gel electrophoresis experiments, the FP signal from HpaII-digested 100% methylated λ DNA remained at the baseline level (Fig 2C). Finally, the assay was used to analyse samples with a known 5mC content according to Formula 1 (Fig 2D). The median global DNA methylation level determined by FP was 0%, 26.6%, 53%, 75.2% and 102.9% for the theoretical 5mC levels 0%, 25%, 50%, 75% and 100%, respectively.

### Application to human genomic DNA

**Fig 3:**
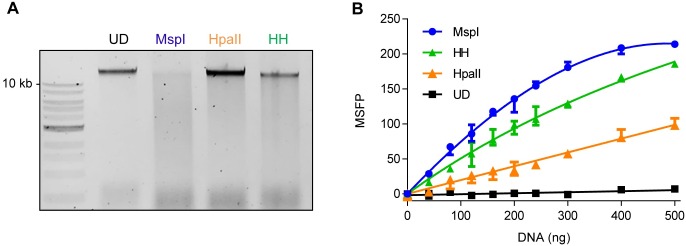
Application of the MSFP assay to human genomic DNA. (A) Digestion pattern of THP-1 genomic DNA digested with MspI, HpaII, or a combination of HpaII and HpyCH4IV (HH). The digested DNA was separated by agarose gel electrophoresis. UD; undigested. (B) Standard curves for THP-1 DNA in the MSFP assay. Data represent medians and ranges of three independent experiments performed in technical duplicates or triplicates. The amounts of digested DNA applied per well are indicated on the x-axis.

Unlike λ DNA, human genomic DNA is predominantly methylated at CpG sites. MspI was able to digest human genomic DNA, whereas HpaII was ineffective and a single large fragment remained, matching the undigested control (Fig 3A). The efficacy of DNA digestion was dramatically improved by the application of HpyCH4IV in addition to HpaII (HH). The results of the MSFP assay agreed with the gel electrophoresis data (Fig 3B). As expected, undigested DNA did not yield an FP signal over a broad concentration range. However, in MspI-digested samples, the FP signal increased in a linear manner and achieved saturation at high DNA concentrations (> 400 ng). The standard curves for HpaII and HH were flatter than the MspI curve which did not reach the signal plateau over the range of concentrations we tested. Within the linear signal range, the MspI standard produced an FP signal four-fold higher than the HpaII standard and ~30% higher than the HH standard. The signal-to-noise ratio of the assay was higher for the HH digest than the HpaII digest when applied to 50–80 ng of human genomic DNA per reaction. The inter-assay coefficient of variation for two independent experiments performed in technical triplicates was 1.5%.

### Comparison of the MSFP and LINE-1 pyrosequencing assays applied to human genomic DNA

The MSFP assay was next used to determine global DNA methylation in an experimental setting and the results were compared with LINE-1 pyrosequencing data obtained from the same samples. LINE-1 is an interspersed retrotransposon with more than 500,000 copies in the human genome [45]. LINE-1 methylation is widely used as a surrogate marker for the global DNA methylation level [34, 45, 46].

In order to obtain DNA controls with a broad methylation range, unmethylated and fully-methylated human genomic DNA controls were prepared using the Repli-g Mini kit (Qiagen) and M.SssI methyltransferase according to Tost and Gut [47]. However, the MSFP assay was unable to detect any signal from the unmethylated genomic DNA control and its gradual dilutions with the fully-methylated genomic DNA control (S2 Fig). Therefore, human genomic DNA samples with different levels of 5mC were generated by treating THP-1 cells with two concentrations of the DNA methyltransferase (DNMT) inhibitors, 5-azacytidine (Aza) or decitabine (DAC).

**Fig 4:**
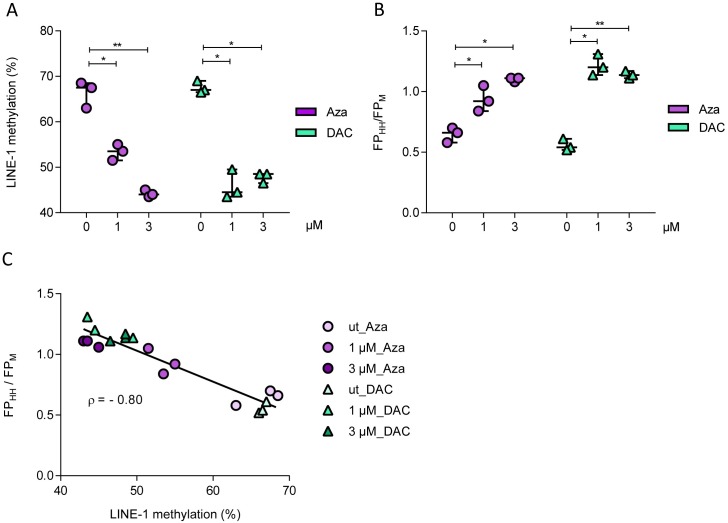
Comparison of the MSFP and LINE-1 assays. (A) Detection of DNA hypomethylation induced by Aza and DAC using the LINE-1 pyrosequencing assay. Three independent experiments were carried out and each data value (circle, triangle) and its median (bar) are shown in a Box-Whisker-Plot. (B) The MSFP assay data present different DNA methylation states as the ratio of the FP_HH_ values to the FP_M_ values. In contrast to the LINE-1 data, a decrease in global DNA methylation (here due to Aza or DAC), results in a higher FP_HH_ value and therefore, in a higher FP_HH_/FP_M_ ratio. The DNA samples used for the MSFP assay were identical to those used for the LINE-1 assay. The data represent three independent experiments with technical triplicates and each data value (circle, triangle) and its median (bar) presented as a Box-Whisker-Plot. The statistical differences between untreated and treated groups (Aza or DAC) were determined using a Kruskall-Wallis test with Dunn’s multiple comparison test (*p<0.05, **p<0.01) for both assays. (C) Correlation between the MSFP and LINE-1 assays. Global DNA methylation level in THP-1 cells treated with DNMT inhibitors (n = 18) was determined using the MSFP and LINE-1 assays, and the reciprocal correlation between the methods determined by two-tailed Spearman’s rank correlation test (ρ = –0.80).

DNA hypomethylation induced by Aza and DAC was detected by LINE-1 pyrosequencing and we observed a significant difference between untreated and corresponding drug-treated groups supported by the Kruskal-Wallis test (Fig 4A). LINE-1 methylation decreased in Aza-treated cells in a concentration-dependent manner, whereas for DAC the 1 μM and 3 μM treatments reduced the methylation to the same extent (45–50%). Global DNA methylation in the Aza and DAC treated THP-1 cells was then analysed using the MSFP assay (Fig 4B). In agreement with the LINE-1 assay, the MSFP assay generated FP values that correlated with the Aza concentration, and significantly higher FP signals were detected in both treated groups at higher concentrations of the test compounds. The significant differences in DNA methylation states between untreated and drug-treated groups were again demonstrated by the Kruskal-Wallis test. The MSFP and LINE-1 results were then compared using Spearman’s rank correlation test (Fig 4C). The Spearman’s rho (ρ) value was –0.80 (95% CI –0.93 to –0.53; p < 0.0001) and the coefficient of determination (ρ^2^) was 0.64 indicating a good correlation between the results obtained with the MSFP and the LINE-1 pyrosequencing assays. Hence, we demonstrated that our MSFP results correlated well with those of the LINE-1 pyrosequencing assay, an established and widely used method for global DNA methylation analysis.

## Discussion

The purpose of this study was to establish a simple and cost-effective approach to the measurement of global DNA methylation, suitable for small as well as large-scale experimental setups, e.g. cell culture-based compound screens. We confirmed the efficacy of the previously established global DNA methylation assay using λ DNA within a broad 5mC range and further improved the assay for human genomic DNA applications by (i) introducing the second methyl-sensitive restriction enzyme HpyCH4IV, already described in the CpGlobal method [19] in addition to HpaII, and (ii) including standard curves to ensure the distribution of FP values of analytes within the linear signal range.

In the context of human genomic DNA, we observed that the slope of the MspI standard curve was notably steeper than that of the HpaII-standard curve, so the input amount of DNA must be limited to a very narrow window, within which FP signals of MspI digests are not saturated but those of HpaII digests are strong enough to generate a sufficient signal-to-noise ratio. It should be noted that both fully- as well as hemi- methylated palindromic CpG sites (dyads) are resistant to HpaII digestion [44, 48], therefore, these are not distinguishable by this assay. Recent analyses revealed variable ranges for CpG-hemimethylation from 1 to 25% (and even higher amounts in methylation mutants) which were dependent on the species, the cell lines and the genetic loci that were analysed [49–52].

The slope of the curve for the HH standard was steep and thus, closer to the MspI standard than the HpaII standard, so it should be possible to obtain FP values with a good signal-to-noise ratio within a linear range using a small amount of DNA. Compared to the original method, which uses various amount of human genomic DNA per reaction (50–500 ng), our assay requires only 50–80 ng [29]. This amount of input DNA is less than that used in most high-throughput global DNA methylation approaches listed in Table 1, making our modification highly sensitive. Even though DNA digestion with the MspI-HpaII pair, which cover 8.04% of human CpG sites, is widely used for epigenetic studies [53], other potential methylation sites beyond its target sequence are overlooked. The combination of restriction enzymes enables researchers to investigate more CpG sites throughout the genome because other studies have successfully improved genome-wide coverage of 5mC by using alternative enzymes or novel enzyme pairs [54, 55].

Unexpectedly, the MSFP assay was unable to detect any signal from samples containing the unmethylated human genomic DNA control generated by the Repli-g kit, although these samples were efficiently digested by both MspI and HH and were applicable in the LINE-1 pyrosequencing assay (S2A and S2C Fig). Accordingly, we presume that components supplied by the kit interfere with the subsequent terminal extension step and/or the subsequent fluorescence detection. However, the MSFP assay was able to detect DNA hypomethylation induced by DNMT inhibitors in good agreement with the LINE-1 pyrosequencing assay and the results of these assays showed significant correlation. There is no direct evidence to show that MSFP assay results agree with other gold standard assays such as high performance liquid chromatography (HPLC), but methyl-sensitive digestion methods have been widely applied in genome-wide methylation studies. A significant correlation between LINE-1 pyrosequencing and HPLC assays has been reported, whereas LUMA, another restriction enzyme-based assay, failed to show a correlation with the HPLC method [31]. A recent study revealed that LUMA and LINE-1 assays yield discordant results, with a significant tissue-specific measurement bias [56]. Currently, MSFP may be useful for the primary screening of compounds that influence global DNA methylation states and these outcomes need to be validated by additional orthogonal assay.

The choice of appropriate high-throughput screening methods depends on several factors (Table 1). Polarization assays are homogeneous, i.e. they do not require the separation of free and bound ligand [57]. MSFP, therefore, allows all reactions, from DNA digestion and terminal extension to the last measurement, to be carried out in a single well on a 384-well plate in less than 3.5 h. The MSFP assay is also advantageous because it features an internal control, i.e. MspI digestion of the DNA in parallel, which prevents the misinterpretation of FP values derived from HpaII or HH digestions that determine the 5mC content of the analytes. Furthermore, MSFP can measure 5mC levels at restriction sites regardless of the species. Finally, we confirmed that FP is highly reproducible and can be automated. MSFP could therefore, be adapted to automated liquid handling platforms, starting with cell culture in 96-well plates followed by direct DNA extraction in a 96-well format, digestion and finally the TAMRA-dCTP incorporation assay.

The most abundant retrotransposon sequences in the human genome are LINE-1 and Alu elements, short interspersed elements that account for ~11% of total genomic DNA [58, 59]. Both elements are widely used as surrogate markers for global DNA methylation. These methods require small amounts of DNA because 5mC residues in the amplified elements are sequenced, although these assays need to be modified to analyse DNA from different organisms. In addition, both LINE-1 and Alu assays require the enrichment of amplicons if pyrosequencing is the downstream method. In addition to MSFP, the LUMA and CpGlobal methods detect 5mC within MspI-HpaII restriction sites. In these approaches, enzymatically digested DNA is terminally extended with dCTPs labelled using fluorophores or biotin, respectively. LUMA directly measures the incorporation of dCTP into restricted DNA loci by pyrosequencing, whereas CpGlobal requires additional rounds of purification, due to the binding of biotin-labelled DNA to the plate and incubation with HRP-conjugated neutravidin for the subsequent luminescence measurement. Compared to these restriction enzyme-based assays, the MSFP requires less DNA and the fluorescence signal can be directly measured on a standard plate reader equipped with polarizing filters. Commercially available ELISA kits can measure the overall level of 5mC in the genome regardless of DNA origin, although these kits are expensive and involve multiple incubation and washing steps that are not suitable for large-scale screening. Importantly, the MSFP is compatible with the 384-well format, whereas all the other approaches are currently restricted to 96-well format instruments.

Finally, the MSFP has potential for use in the diagnosis of the presence or progression of cancer. Global DNA hypomethylation is a hallmark of several tumour types and global DNA methylation states could be used as a biomarker for some malignancies [19, 60–62].

## Conclusions

Our MSFP assay is rapid, simple, sensitive and capable of measuring multiple samples in a single run. Therefore, it offers considerable advantages for a variety of potential applications, including large-scale screening of compounds or reagents that modify DNA methylation states in a global manner and diagnostic use for certain type of malignancies. A significant correlation between the MSFP and the LINE-1 pyrosequencing assay has been demonstrated, although further studies are required to confirm agreement of results obtained by MSFP and the other gold standard assays such as HPLC.

## Materials and Methods

### DNA hypomethylating compounds

We prepared 10 mM stock solutions of 5-azacytidine (Aza, Sigma-Aldrich, St. Louis, MO, USA) and decitabine (DAC, Sigma-Aldrich) in nuclease-free distilled water and stored them at –20°C. Both chemicals inhibit DNA methyltransferase (DNMT) by acting as cytidine analogues [63, 64]. The appropriate concentrations of drugs were freshly prepared in cell culture medium for each treatment.

### In vitro methylation of lambda DNA

Fully methylated bacteriophage lambda (λ) DNA was acquired by incubating 1 μg of λ DNA in a 20-μl reaction containing 1 U of M.SssI methylase, 1X NEBuffer 2, and freshly-prepared 160 μM S-adenosylmethionine at 37°C for 1 h, followed by heat inactivation of the enzyme at 65°C for 20 min (Fig 2). All reagents were purchased from New England BioLabs, Ipswich, United States.

### Cell culture and genomic DNA preparation

Human monocytic cell line, THP-1, was purchased from Deutsche Sammlung von Mikroorganismen und Zellkulturen GmbH (Braunschweig, Germany). The cells were cultured in RPMI-1640 medium-GlutaMAX^TM^ supplemented with 10% (v/v) heat inactivated fetal calf serum and a 1% mixture of penicillin and streptomycin in a humidified incubator containing 5% CO_2_ at 37°C. All reagents mentioned above were purchased from Gibco, Thermo Fisher Scientific (Oberhausen, Germany). Periodically, the cells were controlled for mycoplasma contamination using the MycoAlert^™^ PLUS Mycoplasma Detection Kit (Lonza, Basel, Switzerland). In order to obtain genomic DNA with a broad methylation range, the cells were seeded at a concentration of 0.1 x 10^6^ cells per well in 96-well cell culture plates and were treated with either Aza or DAC (each at two concentrations: 1 and 3 μM) for 72 h. Genomic DNA was extracted using the Agencourt DNAdvance Genomic DNA Isolation Kit (Beckman Coulter, Brea, CA, USA) according to the manufacturer’s instructions (Figs 3 and 4). The use of intact DNA is important to achieve accurate FP and LINE-1 readouts. DNA quality was confirmed by agarose gel electrophoresis.

### Methyl-sensitive fluorescence polarization assay

For the standards, up to 5 μg of DNA was digested using 5 U of MspI, 5 U of HpaII, or a combination of 5 U each of HpaII and HpyCH4IV, in a 100-μl reaction containing 1xCutSmart^®^ Buffer at 37°C for 2 h (Figs 2B and 3B). All enzymes mentioned above were purchased from the New England BioLabs. We also digested λ DNA (10 ng) and genomic DNA (50–80 ng) samples with MspI (0.5 U) or HpaII (0.5 U), or a combination of HpaII and HpyCH4IV (each 0.5 U) in a 10-μl reaction at 37°C for 2 h (Figs 2C, 2D and 4B, and S2B and S2C Fig). Terminal extension of the digested DNA was achieved by incubation with 15 μl of terminal extension buffer containing 1.7 x PCR buffer (34 mM Tris-HCl, pH 8.4, 85 mM KCl), 2.55 mM MgCl_2_, 0.75 U Taq DNA polymerase (Thermo Fisher Scientific), and 17 nM 5-propargylamino-dCTP-5/6-carboxytetramethyl-rhodamine (TAMRA-dCTP; Jena Bioscience, Jena, Germany) in a 384-well black flat-bottom plate (Greiner Bio-One, Kremsmünster, Austria) at 58°C for 1 h in the dark. The incorporation of fluorescent TAMRA-dCTP into digested DNA on the plate was directly measured using the Infinite 200 PRO plate-reader (Tecan, Männedorf, Switzerland) equipped with polarized light-sensitive filters (excitation/emission wavelength: 535/590 nm) and fluorescence polarization values were computed using i-control^™^ software (Tecan). All standards and samples were assayed in technical duplicates or triplicates and the averages and standard deviations were calculated from background-corrected FP values. Standards were included in all experiments to ensure the FP signals fell within the linear signal range. FP values of samples digested with MspI, HpaII or HpaII plus HpyCH4IV (HH) report the amount of DNA and the extent of 5mC, respectively. Therefore, the 5mC content (%) of λ DNA was assessed according to Formula 1 below, and the 5mC content of genomic DNA was calculated as FP_HH_/FP_M_. The FP value of the HH-cleaved sample (FP_HH_) was normalized to the corresponding MspI signal (FP_M_).

$$\lambda \;DNA\;5mC\;methylation\;level\;\left(\%\right)\;\left(0\%\;Standard\;FP_{H}∕FP_{M}-\;Sample\;FP_{H}∕FP_{M}\right)\times 100$$ (1)

### Agarose gel electrophoresis

DNA (100 ng) was digested with MspI (0.5 U), HpaII (0.5 U) or a combination of HpaII and HpyCH4IV (each of 0.5 U) in a 10-μl reaction containing 1xCutsmart^®^ buffer at 37°C for 1 h. Digested DNA was separated by 0.8% agarose gel electrophoresis (80 V, 30 min) on gels pre-stained with Roti-safe (Carl-Roth, Karlsruhe, Germany) and was visualized by ChemiDoc MP (Bio-Rad, Hercules, CA, USA). Acquired images were processed using Image Lab software (Bio-Rad). For each gel electrophoresis, 1 kb DNA Ladder (NEB) was applied onto the gel (Figs 2A and 3A, S1 and S2C Figs).

### LINE-1 pyrosequencing assay

The level of 5mC in long interspersed nuclear element-1 (LINE-1) sequences was investigated as previously described [34, 35]. Briefly, 200–500 ng genomic DNA was bisulfite converted using the EZ-96 DNA Methylation-Lightning Mag Prep Kit (Zymo Research, Irvine, CA, USA) according to the manufacturer’s instructions. A promoter region (L1Hs) of a LINE-1 sequence that covers four CpG sites was amplified in a 50-μl reaction including 5 μl bisulfite-converted DNA, 2.5 U of MyTaq^™^ HS DNA Polymerase (Bioline, London, United Kingdom), 5x MyTaq Reaction Buffer (Bioline), and 0.4 μM of each forward (5'-TTT TGA GTT AGG TGT GGG ATA TA-3') and biotinylated [Btn] reverse (5'-[Btn]AAA ATC AAA AAA TTC CCT TTC-3') primers. Each PCR comprised an initial denaturation step (95°C for 1 min), 40 amplification cycles (95°C for 15 s, 56°C for 15 s, 72°C for 15 s), and a final extension step (72°C for 5 min). In addition, a water control without the DNA template and genomic DNA controls from EpiTech PCR Control DNA set (Qiagen, Hilden, Germany) were included in the PCR. The specificity and quality of the resulting amplicons were confirmed using the Qiaxel DNA Screening Kit (2400) (Qiagen). The amplified LINE-1 sequence was analysed using a sequencing primer (5’-AGT TAG GTG TGG GAT ATA GT-3’) and PyroMark Gold Q96 Reagents (Qiagen) by Pyrosquencing^TM^ (Qiagen) with nucleotide dispensation order (ATC AGT GTG TCA GTC AGT CTA GTC TG) determined by Pyro Q-CpG methylation software v1.0.9. A cytosine located beyond a CpG site served as an internal bisulfite conversion control. Samples with incomplete bisulfite conversion were excluded from analysis. The median was calculated from the methylation percentage across the four analysed CpG sites (Fig 4).

### Statistical analysis

The Graphpad Prism v6 (GraphPad Software, La Jolla, CA, USA) was used to carry out statistical tests. The correlations between assay results were calculated by the Spearman’s rank correlation test, and pairwise group comparisons were performed using the Kruskal-Wallis test with Dunn’s multiple comparison test (Fig 4).

## Supporting Information

S1 Fig In vitro methylation of lambda DNA for LC-MS/MS analysis. Fully-methylated λ DNA was prepared and the complete methylation was confirmed by digestion with either MspI or HpaII, as described in the Materials and Methods section, prior to LC-MS/MS analysis (S1 Table).
(TIF)

S2 Fig Testing unmethylated and fully-methylated human genomic DNA controls. (A) Quantification of global DNA methylation level in human genomic DNA controls using the LINE-1 pyrosequencing assay. Unmethylated (THP-1_UM) and fully-methylated (THP-1_M) human genomic DNA controls were prepared from THP-1-derived DNA using the Repli-g Mini kit (Qiagen) and M.SssI methyltransferase, respectively, as described in the Results section. For comparison, the global DNA methylation level of untreated THP-1 (THP-1) and human genomic DNA controls from EpiTech PCR Control DNA set (Qiagen) were also measured (Q_UM; Qiagen unmethylated DNA control, Q_M; Qiagen methylated DNA control). The data represent medians and ranges of two to three independent experiments. (B) Measurement of 5mC in human genomic DNA controls with varying 5mC content, using the MSFP assay. Human genomic DNA controls with varying DNA methylation states (100 ng) were obtained by mixing THP-1_UM and THP-1_M DNA controls. The data show medians and ranges of two independent experiments. (C) Digestion pattern of the human genomic DNA controls with various 5mC content. Subsequent to MSFP measurement (shown in B), samples from a 384-well plate were collected and loaded on agarose gel.
(TIF)

S1 Table LC-MS/MS analysis of in vitro methylated lambda DNA. Genome-wide 5mC content in unmethylated and in vitro methylated λ DNA, analysed in S1 Fig, was determined using liquid chromatography-tandem mass spectrometry (LC-MS/MS). The detailed protocol is described in S1 Protocol. The λ genome is 48,502 bp in length (NCBI Accession: NC_001416.1). Using Clone Manager 9 Professional Edition (Scientific & Educational Software; Denver, CO, USA) we counted 24,182 2’-deoxy-cytidine (dC) in total per λ genome sequence and detected 3,113 CpG sites corresponding to 6,226 dC which are potentially susceptible to M.SssI methylation. Thus, complete methylation of all CpG sites would result in an amount of 25.7% methylated 2’-deoxy-cytidine (m-dC), which is very close to the amount revealed by LC-MS/MS (25.6%) for the in vitro methylated sample. The purchased λ DNA is isolated from a dcm+ E.coli strain. The amount of Dcm (DNA cytosine methyltransferase) methylation at the CC[A/T]GG site is not further specified (manufacturer's information). Thus, the presence of residual 2’-deoxy-cytidine methylation, detected in the untreated λ DNA (0.3%), is presumably due to Dcm activity.
(TIF)

S1 Protocol LC-MS/MS analysis of in vitro methylated lambda DNA. (DOCX)
