# Supplementary figures and images for: High-Throughput Analysis of Global DNA Methylation Using Methyl-Sensitive Digestion
Source: PLoS One. 2016 Oct 17;11(10):e0163184. doi: 10.1371/journal.pone.0163184 (PMC5066982; doi:10.1371/journal.pone.0163184)

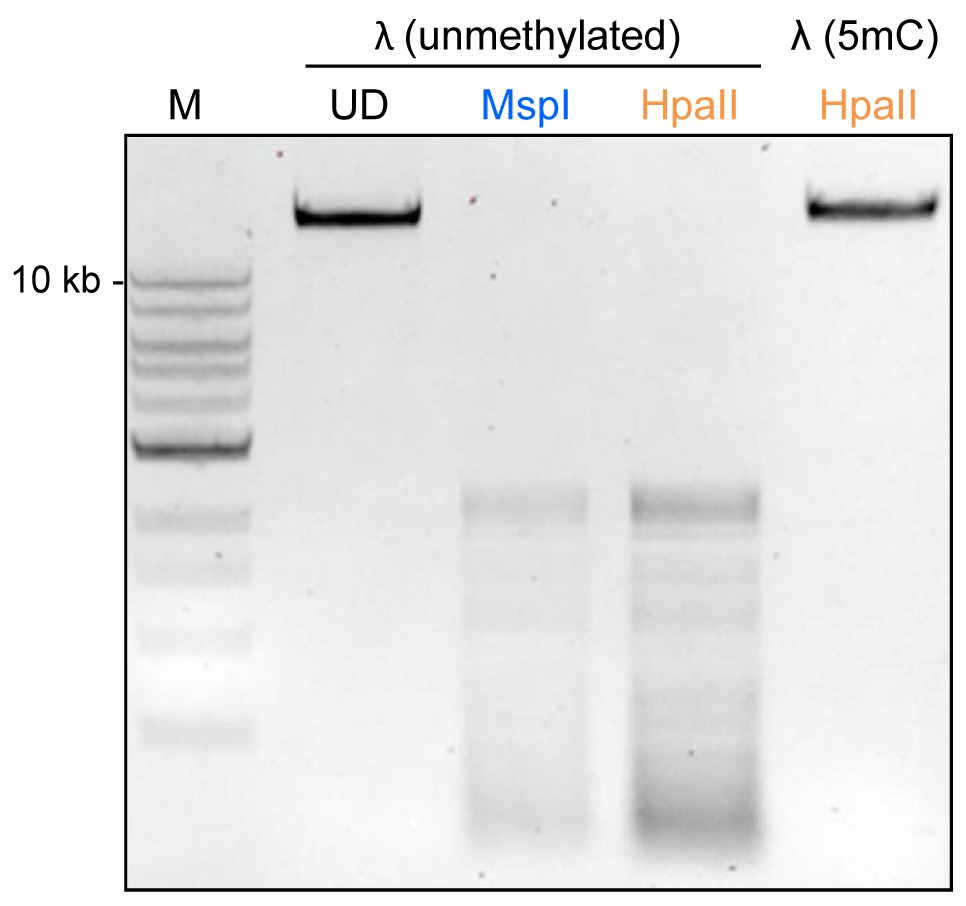

Supplement: S1 Fig — Fully-methylated λ DNA was prepared and the complete methylation was confirmed by digestion with either MspI or HpaII, as described in the Materials and Methods section, prior to LC-MS/MS analysis (S1 Table). (TIF) [file pone.0163184.s001.tif]

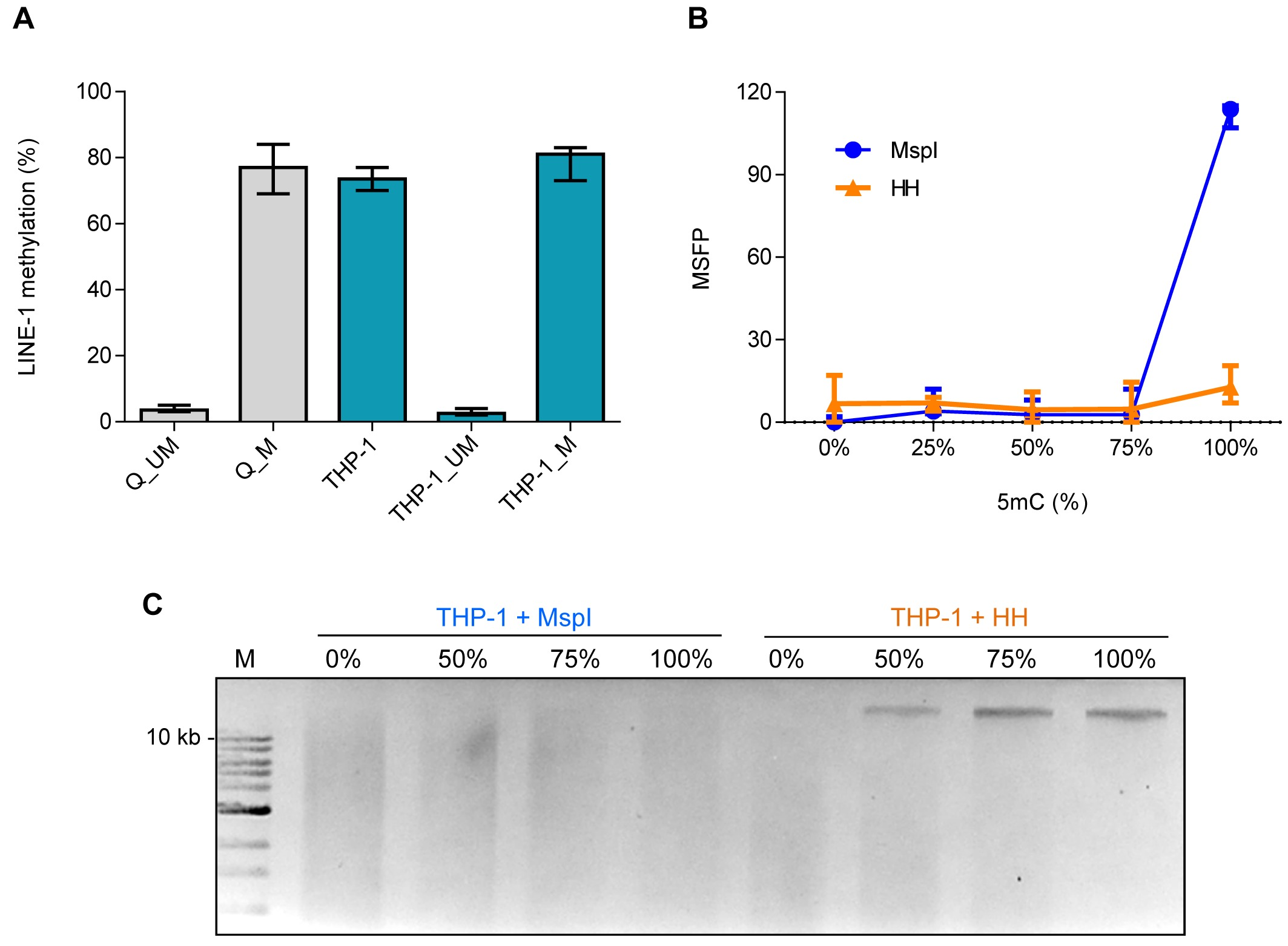

Supplement: S2 Fig — (A) Quantification of global DNA methylation level in human genomic DNA controls using the LINE-1 pyrosequencing assay. Unmethylated (THP-1_UM) and fully-methylated (THP-1_M) human genomic DNA controls were prepared from THP-1-derived DNA using the Repli-g Mini kit (Qiagen) and M.SssI methyltransferase, respectively, as described in the Results section. For comparison, the global DNA methylation level of untreated THP-1 (THP-1) and human genomic DNA controls from EpiTech PCR Control DNA set (Qiagen) were also measured (Q_UM; Qiagen unmethylated DNA control, Q_M; Qiagen methylated DNA control). The data represent medians and ranges of two to three independent experiments. (B) Measurement of 5mC in human genomic DNA controls with varying 5mC content, using the MSFP assay. Human genomic DNA controls with varying DNA methylation states (100 ng) were obtained by mixing THP-1_UM and THP-1_M DNA controls. The data show medians and ranges of two independent experiments. (C) Digestion pattern of the human genomic DNA controls with various 5mC content. Subsequent to MSFP measurement (shown in B), samples from a 384-well plate were collected and loaded on agarose gel. (TIF) [file pone.0163184.s002.tif]

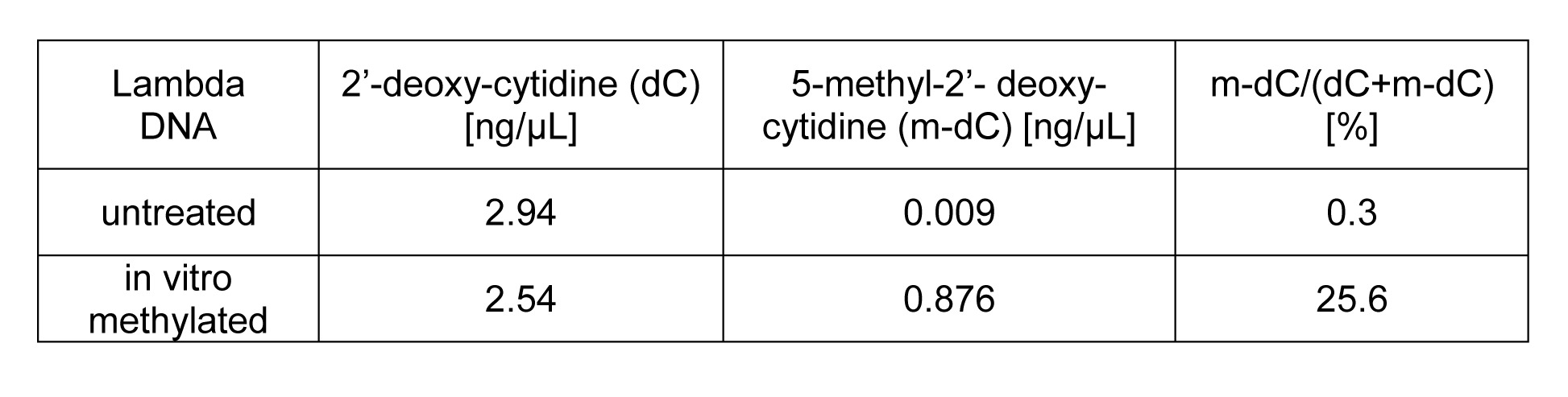

Supplement: S1 Table — Genome-wide 5mC content in unmethylated and in vitro methylated λ DNA, analysed in S1 Fig, was determined using liquid chromatography-tandem mass spectrometry (LC-MS/MS). The detailed protocol is described in S1 Protocol. The λ genome is 48,502 bp in length (NCBI Accession: NC_001416.1). Using Clone Manager 9 Professional Edition (Scientific & Educational Software; Denver, CO, USA) we counted 24,182 2’-deoxy-cytidine (dC) in total per λ genome sequence and detected 3,113 CpG sites corresponding to 6,226 dC which are potentially susceptible to M.SssI methylation. Thus, complete methylation of all CpG sites would result in an amount of 25.7% methylated 2’-deoxy-cytidine (m-dC), which is very close to the amount revealed by LC-MS/MS (25.6%) for the in vitro methylated sample. The purchased λ DNA is isolated from a dcm+ E.coli strain. The amount of Dcm (DNA cytosine methyltransferase) methylation at the CC[A/T]GG site is not further specified (manufacturer's information). Thus, the presence of residual 2’-deoxy-cytidine methylation, detected in the untreated λ DNA (0.3%), is presumably due to Dcm activity. (TIF) [file pone.0163184.s003.tif]
