# Supplementary material for: High-Throughput Analysis of Global DNA Methylation Using Methyl-Sensitive Digestion
Source: PLoS One. 2016 Oct 17;11(10):e0163184. doi: 10.1371/journal.pone.0163184 (PMC5066982; doi:10.1371/journal.pone.0163184)
Supplement: S1 Protocol — (DOCX) [file pone.0163184.s004.docx]

## **S1 Protocol. LC-MS/MS analysis of in vitro methylated lambda DNA**

Untreated and in vitro methylated lambda DNA were treated with DNA Degradase Plus^TM^ (Zymo Research Europe, Irvine, CA, USA) to generate single nucleosides lacking the phosphate residue. Briefly, 500 ng of DNA were treated with 2.5 U DNA Degradase Plus^TM^ enzyme mix in a 25-µl reaction volume at 37°C for 2 h, followed by a heat-inactivation step at 70°C for 20 min. Complete DNA degradation was checked by agarose gel electrophoresis.

The concentrations of unmethylated 2’-deoxy-cytidine (dC) and methylated 2’-deoxy-cytidine (m-dC) were determined using liquid chromatography tandem mass spectrometry (LC-MS/MS). We diluted 10 µl of the degraded lambda DNA solution with 490 µl of methanol. The samples were centrifuged and 50 µl of the supernatant were mixed with 20 µl internal standard (IS) solution containing ^13^C_5_-cytidine (Omicron, South Bend, IN, USA) and ^13^C,^15^N_3_-2'-deoxycytidine (Alsachim, Illkirch, France), each of 100 ng/ml, then evaporated to dryness at 45°C under a slight stream of nitrogen. Just before analysis, the samples were reconstituted in 50 µl of 10 mM acetic acid/ammonium acetate buffer. Calibration standards and quality control samples were prepared identically, but the buffer was replaced by differently concentrated standard solutions.

For chromatographic separation, an Agilent 1260 Series binary pump, degasser and column oven (Agilent Technologies, Waldbronn, Germany) connected to a CTC PAL autosampler (CTC Analytics AG, Zwingen, Switzerland) was used. The analytes were separated using an Atlantis T3 column (100 mm x 2.1 mm I.D., 3 µm particle size and 100 Å pore size; Waters, Milford, MA, USA) in combination with an AQ C18 guard column (4 mm x 2 mm I.D.; Phenomenex, Aschaffenburg, Germany). The temperature of the column oven was set at 40°C, the flow rate was 300 µl/min and the injection volume was 20 µl. The liquid chromatography was carried out with a gradient program using two different mobile phases; eluent A (10 mM acetic acid/ammonium acetate buffer) and eluent B (acetonitrile/water 9:1 with 10 mM acetic acid/ammonium acetate). The gradient program started with 98% eluent A for 2 min, within 4 min the fraction of A was linearly decreased to 60% and remained so for 1 min. For 0.5 min, solvent A was linearly increased again to 98% and the column was re-equilibrated for 3.5 min. The total runtime was 11 min.

A hybrid triple quadrupole – ion trap mass spectrometer 5500 QTRAP (Sciex, Darmstadt, Germany) equipped with a Turbo Ion Spray source operated in positive ion mode was used for detection. The mass spectrometer was operated in positive multiple reaction monitoring mode (MRM) with a dwell time of 50 ms for all precursor-to-product ion transitions. The ionspray voltage was set at 5000 V with an ionization source temperature of 450°C. Ion source gas 1 and 2 were both set at 50 psi while curtain gas was 35 psi and collision gas 9 psi. All quadrupoles were running at unit resolution. The precursor-to-product ion transitions used for quantification were *m/z* 228.1 🡪 112.0 for dC (collision energy 27 V) and *m/z* 242.1 🡪 126.1 for m-dC (collision energy 25 V).

Data was acquired using Analyst Software v1.6 (Sciex) and quantified using MultiQuant Software v3.0 (Sciex) employing the IS method (isotope dilution mass spectrometry). ^13^C_5_-cytidine and ^13^C,^15^N_3_-2'-deoxycytidine were used as IS for m-dC and d-C, respectively. The ratios of analyte peak area and internal standard area (y-axis) were plotted against concentration (x-axis) and calibration curves were calculated by linear regression with 1/x weighting. Calibration ranges were 0.025 – 5.0 ng/ml and 0.005 – 1 ng/ml DNA lysate for dC and m-dC, respectively.
